# Effect of disease-modifying anti-rheumatic drugs on bone structure and strength in psoriatic arthritis patients

**DOI:** 10.1186/s13075-019-1938-3

**Published:** 2019-07-03

**Authors:** David Simon, Arnd Kleyer, Sara Bayat, Koray Tascilar, Eleni Kampylafka, Timo Meinderink, Louis Schuster, Ramona Petrov, Anna-Maria Liphardt, Juergen Rech, Georg Schett, Axel J. Hueber

**Affiliations:** Department of Internal Medicine 3 - Rheumatology and Immunology, Friedrich-Alexander University (FAU) Erlangen-Nürnberg and Universitätsklinikum Erlangen, Ulmenweg 18, 91054 Erlangen, Germany

**Keywords:** Psoriatic arthritis, Bone, Disease-modifying anti-rheumatic drugs, Biological agents, Methotrexate, Computed tomography

## Abstract

**Objectives:**

To address whether the use of methotrexate (MTX) and biological disease-modifying anti-rheumatic drugs (bDMARDs) impacts bone structure and biomechanical properties in patients with psoriatic arthritis (PsA).

**Methods:**

This is a cross-sectional study in PsA patients receiving no DMARDs, MTX, or bDMARDs. Volumetric bone mineral densities (vBMDs), microstructural parameters, and biomechanical properties (stiffness/failure load) were determined by high-resolution peripheral quantitative CT and micro-finite element analysis in the respective groups. Bone parameters were compared between PsA patients with no DMARDs and those receiving any DMARDs, MTX, or bDMARDs, respectively.

**Results:**

One hundred sixty-five PsA patients were analyzed, 79 received no DMARDs, 86 received DMARDs, of them 52 bDMARDs (TNF, IL-17- or IL-12/23 inhibitors) and 34 MTX. Groups were balanced for age, sex, comorbidities, functional index, and bone-active therapy, while disease duration was longest in the bDMARD group (7.8 ± 7.4 years), followed by the MTX group (4.6 ± 7.4) and the no-DMARD group (2.9 ± 5.2). No difference in bone parameters was found between the no-DMARD group and the MTX group. In contrast, the bDMARD group revealed significantly higher total (*p* = 0.001) and trabecular vBMD (*p* = 0.005) as well as failure load (*p* = 0.012) and stiffness (*p* = 0.012). In regression models, age and bDMARDs influenced total vBMD, while age, sex, and bDMARDs influenced failure load and stiffness.

**Conclusion:**

Despite longer disease duration, bDMARD-treated PsA patients benefit from higher bone mass and better bone strength than PsA patients receiving MTX or no DMARDs. These data support the concept of better control of PsA-related bone disease by bDMARDs.

**Electronic supplementary material:**

The online version of this article (10.1186/s13075-019-1938-3) contains supplementary material, which is available to authorized users.

## Introduction

Psoriatic arthritis (PsA) is a chronic inflammatory joint disease associated with psoriasis characterized by peripheral arthritis and enthesitis leading to structural damage [1–3]. Bone erosions and enthesiophytes are hallmarks of local structural damage in PsA. More recently, the impact of PsA on systemic bone is increasingly appreciated. Hence, systemic bone loss has been documented to occur in PsA [4, 5] and increased prevalence of fractures in PsA patients is reported [6].

In contrast to RA, little is known about the effect of disease-modifying anti-rheumatic drugs (DMARDs) on bone structure in PsA. In rheumatoid arthritis (RA), biological DMARDs (bDMARDs) have shown to inhibit bone loss and thus may prevent pathological fractures [7–9]. To date, no such studies have been done in PsA; however, it can be assumed that effective control of inflammation may also impact secondary bone loss and bone biomechanics in PsA patients. In support of this notion, bDMARD treatment in PsA patients retards the progression of periarticular bone erosions [10–12] and periarticular bone loss [13–15]. Whether systemic bone mass and bone biomechanical properties are influenced by DMARD treatment is unclear to date. Furthermore, methotrexate treatment may not necessarily share potential beneficial effects of bDMARDs on bone in PsA patients.

While dual-energy X-ray absorptiometry (DXA) or digital X-ray radiogrammetry (DXR) can quantify bone loss, they do not allow separate assessment of changes in the cortical and trabecular bone compartment or the biomechanical properties of bone. High-resolution peripheral quantitative CT (HR-pQCT) enables the analysis of bone mass and microstructure and via integration of micro-finite element analysis (μFEA) the assessment of bone strength [16]. An HR-pQCT study in RA patients has already shown that the biomechanical properties of the bone are reduced in RA patients [17].

To investigate whether DMARD treatment influences systemic bone structure and function in PsA patients, we investigated bone density, bone microstructure, and biomechanical properties in patients receiving either no DMARDs, methotrexate (MTX) treatment, or biologic DMARDs (bDMARDs) by HR-pQCT. In this cross-sectional study, we were specifically interested, whether PsA patients treated with either MTX or bDMARDs show a better bone structure and function than PsA patients receiving no DMARDs.

## Methods

### Psoriatic arthritis patients

PsA patients were part of the Erlangen Imaging Cohort (ERIC), which prospectively assesses bone composition in patients with inflammatory arthritis [18]. All participants were consecutively recruited during routine diagnostic assessments at the Department of Internal Medicine 3 of the University of Erlangen-Nuremberg. Patients were examined by experienced rheumatologists (AK, JR, AJH) and had to fulfill the Classification criteria for Psoriatic Arthritis (CASPAR) [19]. Age, sex, and smoking habits were recorded. With respect to psoriatic disease, duration of PsA, duration of psoriasis, minimal disease activity (MDA) state, disease activity in psoriatic arthritis (DAPSA), Psoriasis Area and Severity Index (PASI), dermatology life quality index (DLQI), health assessment questionnaire (HAQ), nail and/or scalp involvement, anti-rheumatic and bone-active medication, and laboratory parameters (rheumatoid factor, anti-citrullinated protein antibodies) were collected. Patients were categorized into three different treatment groups: (A) methotrexate monotherapy (over at least 6 months), (B) bDMARDs (without MTX) including TNF inhibitors, secukinumab and ustekinumab (over at least 6 months), and (C) a control group of PsA patients receiving no DMARDs over the last 6 months. The no-DMARD group included patients that for different reasons were not on DMARDs including (i) treatment-naïve patients at their first visit; (ii) treatment-naïve patients who had mild disease that was not considered to require immediate DMARD treatment; (iii) DMARD pre-exposed patients that had stopped treatment for compliance, intolerance, or lack of efficacy; and (iv) patients that were in drug-free remission. The study was conducted on approval of the local ethics committee of the University Clinic of Erlangen. Each individual provided informed consent.

### HR-pQCT measurement

HR-pQCT was performed at the distal radius (dominant hand) by an XtremeCT scanner (Scanco Medical, Brüttisellen, Switzerland). The following bone parameters were assessed: volumetric bone mineral density (vBMD) of the total, trabecular, meta-trabecular, inner trabecular, and cortical bone (all: mg HA/cm^3^) and ratio of meta-to-inner density (%) and cross-sectional bone area (mm^2^). Bone microstructure was assessed by determining trabecular bone volume fraction (%), trabecular number (1/mm), thickness (mm), separation (mm), network inhomogeneity (SD of 1/trabecular number (mm)), and cortical thickness (mm) [20–22].

### Micro-finite element analysis

For micro-finite element analysis (μFEA), finite element analysis software (FAIM, version 8.0, Numerics88 solution, Calgary, Canada) was used. In order to generate micro-finite element models, the segmented trabecular network and cortex of the HR-pQCT images were used [23]. Mesh size of the resulting models ranged from 1.5 to 3.5 million equally sized brick elements. Single linear isotropic tissue modeling was applied by assigning a tissue modulus of 6829 MPa and a Poisson’s ratio of 0.3 homogeneously to each element [16]. A linear uniaxial compression test was simulated. Nodes on the proximal bone surface were fixed in *z* direction but unconstrained in *x* and *y* directions. Nodes on the distal bone surface were also free in the *x* and *y* directions but exposed to a displacement equivalent to 1% strain along the *z* axis [16]. Axial bone stiffness (kN/mm) as reaction force (RFz) divided by average displacement of the distal surface (Uz) and bone strength as estimated failure load (N) based on the Pistoia criterion was calculated [24].

### Statistical analysis

Statistical analyses compared PsA patients treated by bDMARDs or methotrexate to patients receiving no DMARD with respect to vBMD, bone microstructure, and biomechanical properties. Categorical variables are presented as numbers and percentages, and continuous variables as mean ± SD. Frequency distributions of categorical variables were compared using *χ*^2^ tests. Clinical, bone structural, and μFEA parameters were compared by using Kruskal-Wallis test (KW) with subsequent pairwise Mann-Whitney *U* tests, if KW test was significant. In order to account for multiple testing, we applied Bonferroni-Holm adjustment for pairwise comparisons. Critical *p* values for adjusted levels of significance are shown in the corresponding tables. Finally, we fitted linear regression models to contrast the differences in HR-pQCT and μFEA measurements with methotrexate and bDMARD use in comparison to no DMARDs. Models were adjusted for age, gender, and gender-treatment interaction. As a sensitivity analysis, we re-ran the models including MDA. All data manipulation and analyses were conducted using R (V3.5.1, R Foundation for Statistical Computing, Vienna, Austria.).

## Results

### Characteristics of psoriatic arthritis patients

One hundred sixty-five PsA patients were included, 86 of them received DMARD treatment. Thirty-four PsA patients received MTX, and 52 bDMARDs for at least 6 months. Within the bDMARD group, 31 patients had TNF inhibitors (13 adalimumab, 6 infliximab, 9 etanercept, 2 certolizumab, and 1 golimumab), 16 patients received the IL-17A inhibitor secukinumab, and 5 patients the IL12/23 inhibitor ustekinumab. The mean duration of bDMARD treatment was 3.9 ± 3.3 years. Eleven patients in the bDMARD group had received previous MTX, while no bDMARD patient per definition received concurrent MTX. Seventy-nine PsA patients serving as the control group received no DMARDs. Detailed information on the demographic and disease-specific characteristics of the patients is shown in Table 1. Briefly, age and sex distribution, functional index, and comorbidities were not different among the no-DMARD, MTX, and bDMARD groups. The use of bone-active treatments such as vitamin D and anti-resorptive drugs was also balanced. A greater proportion of bDMARD-treated PsA patients received glucocorticoids compared to the no-DMARD group (*p* = 0.002). In addition, a larger proportion of patients under bDMARD therapy had received glucocorticoids in the past (*p* = 0.001) and hence had a longer duration of glucocorticoid therapy.

**Table 1:** Demographic and disease-specific characteristics in the three treatment subgroups

	No DMARDs, *N* = 79	bDMARD, *N* = 52	Methotrexate, *N* = 34	*p* value a/b/c
Demographic characteristics				
Sex (M/F)	31/48	29/23	17/17	−/−/−
Age, (mean ± SD)	49.3 ± 12.0	48.0 ± 11.7	50.9 ± 11.3	−/−/−
Body mass index, (mean ± SD)	28.6 ± 6.2	29.0 ± 6.0	28.8 ± 6.4	−/−/−
Smokers, *N* (%)	24 (30)	12 (23)	7 (21)	−/−/−
Menopause, *N* (%)	21 (27)	12 (23)	7 (21)	−/−/−
Previous fracture, *N* (%)^‡^	4 (5)	2 (4)	2 (6)	−/−/−
Disease-specific characteristics				
Duration of PSO (years), (mean ± SD)	18.1 ± 16.1	20.2 ± 11.1	20.3 ± 16.4	−/−/−
Duration of PsA (years), (mean ± SD)	2.9 ± 5.2	7.8 ± 7.4	4.6 ± 7.4	< *0.001*/*0.011*/*0.018*
MDA, *N* (%)	28 (35)	25 (48)	22 (65)	−/*0.004*/*−*
DAPSA				
DAPSA score, (mean ± SD)	17.0 ± 11.4	12.3 ± 9.6	15.8 ± 17.8	*0.009*/−/−
Remission, *N* (%)	7 (9)	10 (19)	6 (18)	−/−/−
Low activity, *N* (%)	29 (37)	25 (48)	13 (38)	−/−/−
Moderate activity, *N* (%)	24 (30)	12 (23)	9 (27)	−/−/−
High activity, *N* (%)	12 (15)	2 (4)	4 (12)	0.040/−/−
Nail involvement, *N* (%)	20 (25)	7 (14)	8 (24)	−/−/−
Scalp involvement, *N* (%)	35 (44)	8 (15)	8 (24)	*0.002*/−/−
PASI (units), (mean ± SD)	4.0 ± 4.7	1.2 ± 2.7	2.1 ± 7.9	< *0.001*/*0.001*/−
DLQI (units), (mean ± SD)	7.5 ± 6.5	3.2 ± 5.3	6.0 ± 5.3	0.043/−/−
HAQ (units), (mean ± SD)	0.6 ± 0.5	0.5 ± 0.5	0.7 ± 0.8	−/−/−
Diabetes mellitus, *N* (%)	2 (3)	3 (6)	2 (6)	−/−/−
Hypertension, *N* (%)	19 (24)	14 (27)	5 (15)	−/−/−
Autoantibody status				
Positive low-titer ACPA, *N* (%)*	1 (1)	0	1 (3)	−/−/−
Positive low-titer RF, *N* (%)**	3 (4)	0	1 (3)	−/−/−
Anti-rheumatic and bone treatments				
Vitamin D supplementation, *N* (%)	10 (13)	11 (21)	9 (27)	−/−/−
Bisphosphonates, *N* (%)	0	1 (2)	0	−/−/−
Current glucocorticoids, *N* (%)	0	6 (12)	5 (15)	*0.002*/*0.001*/−
Former glucocorticoids intake, *N* (%)	4 (5)	13 (25)	5 (15)	*0.001*/−/−
Duration of glucocorticoids intake (years), (mean ± SD)	1.1 ± 1.0	2.8 ± 2.2	0.4 ± 0.2	−/−/0.034

Bonferroni-Holm adjustment: critical *p* values indicating significant results (italicize *p* values) for all investigated parameters were as follows: *p*_1_ = 0.0167, *p*_2_ = 0.025, *p*_3_ = 0.05

*ACPA* anti-citrullinated protein antibody, *bDMARDs* biologic disease-modifying anti-rheumatic drugs, *N* number, *PASI* Psoriasis Area and Severity Index, *PsA* psoriatic arthritis, *PSO* psoriasis, *MDA* minimal disease activity, *DAPSA* Disease Activity Index for Psoriatic Arthritis, *DLQI* Dermatology Life Quality Index, *HAQ* health assessment questionnaire, *RF* rheumatoid factor, *a* no therapy vs. bDMARD, *b* no therapy vs. methotrexate, *c* bDMARD vs. methotrexate

*< 20 U/mL; **> 50 IE/mL

^‡^Fracture in adult life that occured spontaneously, or fractures caused by trauma that would not have led to a fracture in a healthy person

a no therapy vs. bDMARD

b no therapy vs. Methotrexate

c bDMARD vs. methotrexate

bDMARD-treated PsA patients had also the longest disease duration (7.6 ± 8.4 years; *p* < 0.001 compared to no DMARDs) followed by the MTX group (4.6 ± 7.4) and the no-DMARD group (2.9 ± 5.2). With respect to disease control, more of the patients receiving bDMARDs (48%) and MTX (65%) were in the MDA state than those in the no-DMARD group (35%).

### Better bone microstructure and functional properties in PsA patients taking DMARDs

We first compared the no-DMARD control group with PsA patients taking any DMARDs (MTX or bDMARDs). DMARD patients had higher total vBMD (312 ± 53 vs. 290 ± 54, *p* = 0.004) and trabecular vBMD (171 ± 38 vs. 156 ± 39, *p* = 0.010) compared to no-DMARD controls. In addition, they had better bone microstructure indicated by higher number of trabeculae (2.09 ± 0.33 vs. 1.99 ± 0.35, *p* = 0.047), lower trabecular separation (0.43 ± 0.11 vs. 0.47 ± 0.18, *p* = 0.025), and higher cortical thickness (0.77 ± 0.17 vs. 0.71 ± 0.16, *p* = 0.0012). Regarding biomechanical properties, patients receiving DMARDs had higher stiffness and failure load (stiffness, 50.0 ± 15.0 vs. 45.2 ± 13.7, *p* = 0.034; failure load, 2385 ± 687 vs. 2154 ± 621, *p* = 0.026).

### Better bone microstructure and functional properties is confined to PsA patients taking bDMARDs

**Fig. 1:**
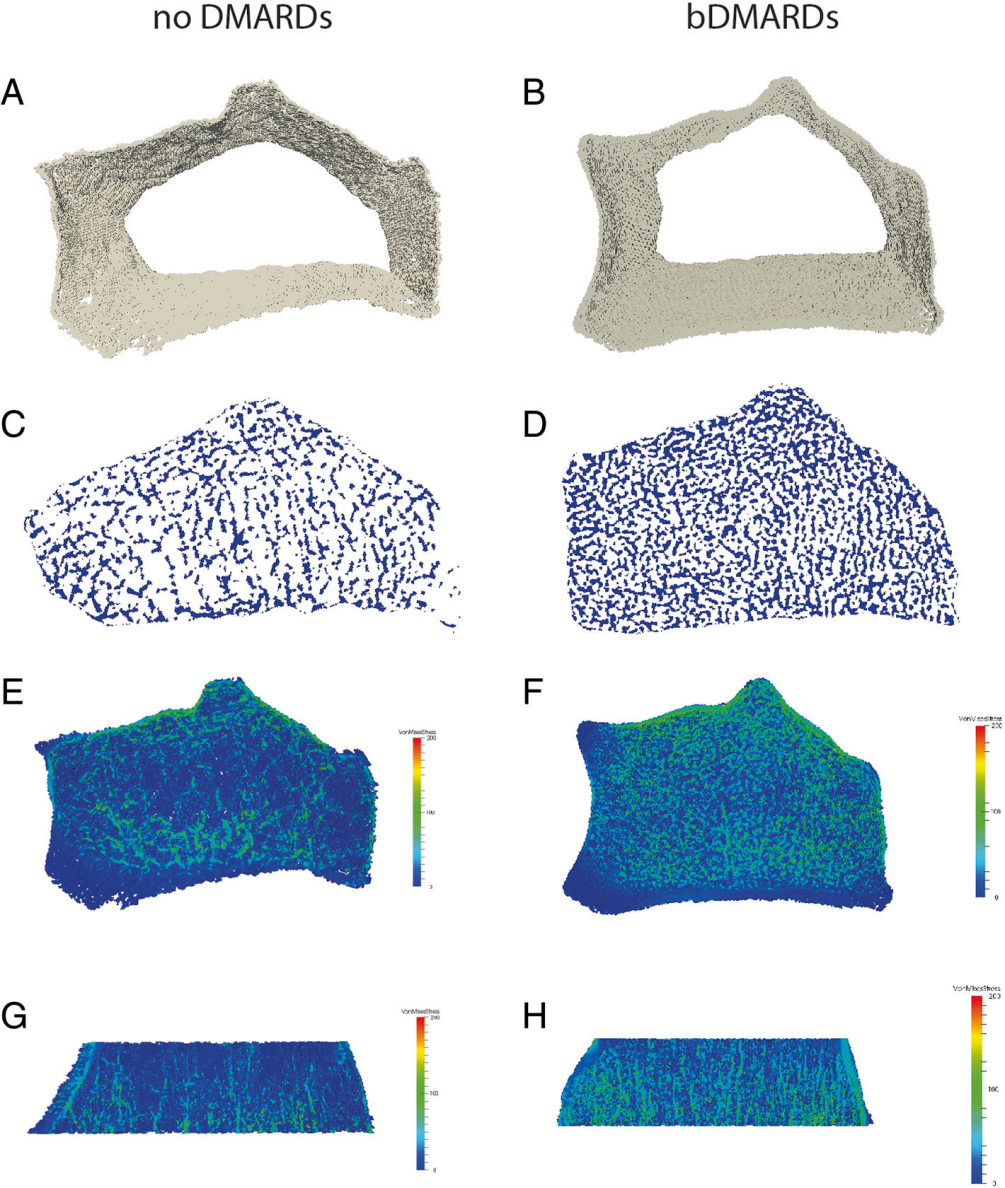
HR-pQCT and finite element analysis. (Left) Psoriatic arthritis (PsA) patient without disease-modifying anti-rheumatic drugs (DMARDs); (right) PsA patient under biological DMARD treatment. Both patients had the same age and sex. **a**, **b** Axial view of three-dimensional reconstruction of the cortical bone. **c**, **d** Axial view of the trabecular bone. **e**, **f** Axial view of finite element analysis-derived stress distribution (full μFEA models). **g**, **h** Coronal view of finite element analysis-derived stress distribution. Colors in **e**–**h** depict von Mises stress (MPa) for the described loading scenario

To test whether the observed better bone status of DMARD-treated PsA patients is based on MTX or bDMARD treatment, we compared bone parameters between the no-DMARD control group and the MTX or the bDMARD group, respectively. The results demonstrated that MTX had no influence on bone microstructure and functional properties (Table 2). In contrast, and despite their longer disease duration, the bDMARD group exhibited significantly higher total and trabecular vBMD (320 ± 44 vs. 290 ± 54, *p* = 0.001; 174 ± 36 vs. 156 ± 39, *p* = 0.005) as compared to the no-DMARD group. In addition, higher cortical thickness (0.80 ± 0.15 vs. 0.71 ± 0.16, *p* = 0.001), with numerically higher numbers and thicker trabeculae (2.12 ± 0.32 vs. 1.99 ± 0.35, *p* = 0.022; 0.069 ± 0.010 vs. 0.065 ± 0.010, *p* = 0.030 (the adjusted significance was not met) were observed in the bDMARD group. Furthermore, also the biomechanical properties of bone (stiffness, 52.1 ± 15.0 vs. 45.2 ± 13.7, *p* = 0.012; failure load, 2473 ± 704 vs. 2154 ± 621, *p* = 0.012) were better in the bDMARD group than in the no-DMARD group (Table 2, Fig. 1).

**Table 2:** Comparison of bone structure and biomechanical properties in no-DMARD-, methotrexate-, and bDMARD-treated PsA patients

	No DMARDs (*N* = 79)	Methotrexate (*N* = 34)	bDMARDs (*N* = 52)	*p* value a/b
Finite element analysis				
Stiffness (kN/mm), (mean ± SD)	45.2 ± 13.7	46.7 ± 14.2	**52.1 ± 15.0** ^**b**^	−/**0.012**
Failure load (N), (mean ± SD)	2154 ± 621	2242 ± 645	**2473 ± 704** ^**b**^	−/**0.012**
Bone parameters				
Volumetric bone mineral density				
Dtotal mg HA/cm^3^, (mean ± SD)	290 ± 54	299 ± 63	**320 ± 44** ^**b**^	−/**0.001**
Dtrab, mg HA/cm^3^, (mean ± SD)	156 ± 39	166 ± 40	**174 ± 36** ^**b**^	−/**0.005**
Dmeta, mg HA/cm^3^, (mean ± SD)	214 ± 38	222 ± 39	**236 ± 35** ^**b**^	−/**0.001**
Dinn, mg HA/cm^3^, (mean ± SD)	116 ± 41	127 ± 43	**132 ± 40** ^**b**^	−/0.026
Dcomp, mg HA/cm^3^, (mean ± SD)	817 ± 57	817 ± 72	831 ± 43	−/−
Meta/Inn, %, (mean ± SD)	1.9 ± 0.5	1.9 ± 0.8	2.0 ± 0.8	−/−
Bone microstructure				
BV/TV, %, (mean ± SD)	0.13 ± 0.03	0.14 ± 0.03	**0.15 ± 0.03** ^**b**^	−/**0.005**
Tb.N, 1/mm, (mean ± SD)	1.99 ± 0.35	2.05 ± 0.36	**2.12 ± 0.32** ^**b**^	−/0.022
TbTh, mm, (mean ± SD)	0.065 ± 0.010	0.067 ± 0.011	**0.069 ± 0.010** ^**b**^	−/0.030
Tb.Sp, mm, (mean ± SD)	0.47 ± 0.18	0.44 ± 0.10	**0.42 ± 0.11** ^**b**^	−/**0.010**
Tb.1/N.SD, mm, (mean ± SD)	0.21 ± 0.16	0.19 ± 0.08	**0.19 ± 0.13** ^**b**^	−/0.017
Ct.Th, mm, (mean ± SD)	0.71 ± 0.16	0.73 ± 0.18	**0.80 ± 0.15** ^**b**^	−/**0.001**
Bone area				
Cross-sectional area (mean ± SD)	321 ± 76	335 ± 88	325 ± 80	−/−

Bonferroni-Holm adjustment: critical *p* values indicating significant results (bold *p* values) for all investigated parameters were as follows: *p*_1_ = 0.0167, *p*_2_ = 0.025, *p*_3_ = 0.05

*bDMARDs* biologic disease-modifying anti-rheumatic drugs, *Dtotal* total vBMD, *Dtrab* trabecular vBMD, *Dcomp* compact vBMD, *Dmeta* meta trabecular vBMD, *Dinn* inner trabecular vBMD, *meta/inn* ratio of meta-to-inner density, *BV/TV* trabecular bone volume fraction, *Tb.N* number of trabeculae, *Tb.Th* trabecular thickness, *Tb.Sp* trabecular separation, *Tb.1/N.SD* inhomogeneity of network, *Ct.Th* cortical thickness, *a/b: a* no DMARDs vs. methotrexate, *b* no DMARDs vs. bDMARD

### Bone structure and function in PsA patients depend on age, sex, bDMARD therapy, and disease activity state

To test the impact of bDMARD treatment on bone structure and function, we set up three regression models with total vBMD, stiffness, and failure load as the respective outcome variable. We found that age and bDMARD treatment had a significant impact on vBMD, while age, sex, and bDMARD treatment influenced the model stiffness and failure load (Table 3). Use of bDMARD treatment, despite the longer disease duration and higher proportion of corticosteroid use, was associated with better bone density and higher stiffness and failure load estimates while no such association was observed with the use of methotrexate in comparison to no-DMARD use. When adding minimal disease activity (MDA) to the regression models, results remained robust (Additional file 1: Table S1).

**Table 3:** Regression models

	Estimates	CI	*p* value
Total vBMD			
Intercept	362.37	325.84–398.90	< *0.001*
Age	− 1.57	− 2.23 to − 0.91	< *0.001*
Female	9.09	− 13.47–31.65	0.427
bDMARDs	43.52	18.18–68.86	*0.001*
Methotrexate	12.12	− 17.47–41.71	0.420
Stiffness			
Intercept	68.65	60.90–76.40	< *0.001*
Age	− 0.28	− 0.42 to − 0.14	< *0.001*
Female	− 15.66	− 20.51 to − 10.82	< *0.001*
bDMARDs	6.81	1.43–12.18	*0.013*
Methotrexate	− 0.67	− 7.06–5.72	0.835
Failure load			
Intercept	3227.06	2879.26–3574.86	< *0.001*
Age	− 12.77	− 19.06 to − 6.49	< *0.001*
Female	− 732.60	− 949.97 to − 515.22	< *0.001*
bDMARDs	322.23	80.84–563.63	*0.009*
Methotrexate	− 4.88	− 291.73–281.97	0.973

Reference for the change is the no treatment group. Models are adjusted for age, gender, and treatment-gender interaction

*vBMD* volumetric bone mineral density, *bDMARDs* biologic disease-modifying anti-rheumatic drugs, *CI* confidence interval

## Discussion

The results of this study reveal that PsA patients receiving bDMARDs show better bone microstructure and biomechanical properties as compared to PsA patients receiving no DMARD treatment. In contrast, such differences are not found in PsA patients receiving MTX treatment suggesting that the beneficial bone effect of DMARD treatment in PsA is confined to the use of bDMARDs. These data are remarkable since bDMARD-treated PsA patients are a selectively more active and more resistant patient population, including a higher glucocorticoid use, which would reflect a higher burden of disease on the bone. However, we found the bone structure in bDMARD-treated PsA patients is not worse but even better that in the control population comprising patients with mild disease indicating a specific beneficial effect of bDMARDs on the bone. Notably, this effect was observed despite significantly longer disease duration in the bDMARD group.

These observations may be explained by previous functional data showing that the two central pro-inflammatory mediators in PsA, IL-17 and TNF, trigger an imbalance in bone homeostasis, increasing osteoclast-medicated bone resorption and inhibiting osteoblast-medicated bone formation [25–28]. This concept of cytokine-mediated bone loss in PsA is in fact supported by this study showing that PsA patients treated with either IL-17 inhibitor or TNFa inhibitors show better structural and functional bone data. We did not find significant differences in bone structure and function between IL-17 inhibitor- and TNFa inhibitor-treated PsA patients, suggesting a similar impact of the two main bDMARD treatment strategies on systemic bone in PsA.

Methotrexate monotherapy did not impact bone structure or function in PsA patients. Values for bone mass, microstructure, and function were consistently in the range of no-DMARD controls. This finding is interesting since the control of signs and symptoms of PsA was similar in the MTX- and bDMARD-treated groups. Hence, indirect effects such as better control of inflammation by bDMARDs are less likely to attribute for these differences. This notion is also supported by the fact that the inclusion of minimal disease activity in the regression models did not affect the results. On the other hand, methotrexate does not seem to share the positive effects of cytokine blockade on the bone. Hence, methotrexate has shown to inhibit osteoblast differentiation and bone formation [29], preventing that the anti-inflammatory effects of MTX are accompanied by sufficient rebalancing of the disturbed bone homeostasis.

A limitation of this study is the fact that it is cross-sectional and not longitudinal. Thus, we cannot conclude that bDMARDs increase bone mass and/or improve bone biomechanics in individual patients. Nonetheless, considering the longer disease duration of DMARD-treated than naïve PsA patients and the preferential use of bDMARDs in more severe PsA cases would suggest more severe rather than milder bone disease in bDMARD-treated patients. Since the exact opposite outcome was observed, the data support a direct effect of bDMARDs on bone structure and function in PsA. Furthermore, patient groups were balanced for several factors that could influence the bone including age and sex and comorbidities like diabetes mellitus, which have recently been shown to influence bone structure in PsA [30] as well as bone-active therapies. As a next step, a longitudinal study based on repeated HR-pQCT measurements will be necessary to better understand the effects of individual bDMARDs on bone homeostasis in PsA.

## Conclusions

In summary, this study shows that the use of bDMARDs is associated with better bone structure and function in PsA patients. Given that PsA is associated with increased fracture risk, fast and adequate neutralization of the key pro-inflammatory and bone-destructive mediators seems to be important to restore bone health and to limit fracture risk in PsA patients.

## Additional file

Additional file 1: **Table S1.** Regression models. (DOCX 18 kb)

## Data Availability

All data generated or analyzed during this study are included in this published article.

## Abbreviations

bDMARDs: Biological disease-modifying anti-rheumatic drugs
CASPAR: Classification criteria for Psoriatic Arthritis
DAPSA: Disease activity in psoriatic arthritis
DLQI: Dermatology life quality index
DMARDs: Disease-modifying anti-rheumatic drugs
DXA: Dual-energy X-ray absorptiometry
DXR: Digital X-ray radiogrammetry
HAQ: Health assessment questionnaire
HR-pQCT: High-resolution peripheral quantitative computed tomography
IL-17: Interleukin-17
KW: Kruskal-Wallis test
MDA: Minimal disease activity
mg HA/cm^3^: Milligram hydroxyapatite/cubic centimeter
MTX: Methotrexate
PASI: Psoriasis Area Severity Index
PsA: Psoriatic arthritis
TNF: Tumor necrosis factor
vBMD: Volumetric bone mineral density
μFEA: Micro-finite element analysis

## References

[1] Schett G, Lories RJ, D'Agostino MA, Elewaut D, Kirkham B, Soriano ER (2017). Enthesitis: from pathophysiology to treatment. Nat Rev Rheumatol.

[2] Ritchlin CT, Colbert RA, Gladman DD (2017). Psoriatic arthritis. N Engl J Med.

[3] Mease PJ (2015). Biologic therapy for psoriatic arthritis. Rheum Dis Clin N Am.

[4] Attia EA, Khafagy A, Abdel-Raheem S, Fathi S, Saad AA (2011). Assessment of osteoporosis in psoriasis with and without arthritis: correlation with disease severity. Int J Dermatol.

[5] Kocijan R, Englbrecht M, Haschka J, Simon D, Kleyer A, Finzel S (2015). Quantitative and qualitative changes of bone in psoriasis and psoriatic arthritis patients. J Bone Miner Res.

[6] Ogdie A, Harter L, Shin D, Baker J, Takeshita J, Choi HK (2017). The risk of fracture among patients with psoriatic arthritis and psoriasis: a population-based study. Ann Rheum Dis.

[7] Hoff M, Kvien TK, Kalvesten J, Elden A, Kavanaugh A, Haugeberg G (2011). Adalimumab reduces hand bone loss in rheumatoid arthritis independent of clinical response: subanalysis of the PREMIER study. BMC Musculoskelet Disord.

[8] Vis M, Voskuyl AE, Wolbink GJ, Dijkmans BA, Lems WF (2005). Bone mineral density in patients with rheumatoid arthritis treated with infliximab. Ann Rheum Dis.

[9] Krieckaert CL, Nurmohamed MT, Wolbink G, Lems WF (2013). Changes in bone mineral density during long-term treatment with adalimumab in patients with rheumatoid arthritis: a cohort study. Rheumatology (Oxford).

[10] Kavanaugh A, Ritchlin C, Rahman P, Puig L, Gottlieb AB, Li S (2014). Ustekinumab, an anti-IL-12/23 p40 monoclonal antibody, inhibits radiographic progression in patients with active psoriatic arthritis: results of an integrated analysis of radiographic data from the phase 3, multicentre, randomised, double-blind, placebo-controlled PSUMMIT-1 and PSUMMIT-2 trials. Ann Rheum Dis.

[11] van der Heijde D, Kavanaugh A, Gladman DD, Antoni C, Krueger GG, Guzzo C (2007). Infliximab inhibits progression of radiographic damage in patients with active psoriatic arthritis through one year of treatment: results from the induction and maintenance psoriatic arthritis clinical trial 2. Arthritis Rheum.

[12] van der Heijde D, Landewe RB, Mease PJ, McInnes IB, Conaghan PG, Pricop L (2016). Brief report: secukinumab provides significant and sustained inhibition of joint structural damage in a phase III study of active psoriatic arthritis. Arthritis Rheumatol.

[13] Hoff M, Kavanaugh A, Haugeberg G (2013). Hand bone loss in patients with psoriatic arthritis: posthoc analysis of IMPACT II data comparing infliximab and placebo. J Rheumatol.

[14] Kampylafka E, d'Oliveira I, Linz C, Lerchen V, Stemmler F, Simon D (2018). Resolution of synovitis and arrest of catabolic and anabolic bone changes in patients with psoriatic arthritis by IL-17A blockade with secukinumab: results from the prospective PSARTROS study. Arthritis Res Ther.

[15] Szentpetery A, McKenna MJ, Murray BF, Ng CT, Brady JJ, Morrin M (2013). Periarticular bone gain at proximal interphalangeal joints and changes in bone turnover markers in response to tumor necrosis factor inhibitors in rheumatoid and psoriatic arthritis. J Rheumatol.

[16] Macneil JA, Boyd SK (2008). Bone strength at the distal radius can be estimated from high-resolution peripheral quantitative computed tomography and the finite element method. Bone..

[17] Stemmler F, Simon D, Liphardt AM, Englbrecht M, Rech J, Hueber AJ (2018). Biomechanical properties of bone are impaired in patients with ACPA-positive rheumatoid arthritis and associated with the occurrence of fractures. Ann Rheum Dis.

[18] Simon D, Kleyer A, Englbrecht M, Stemmler F, Simon C, Berlin A (2018). A comparative analysis of articular bone in large cohort of patients with chronic inflammatory diseases of the joints, the gut and the skin. Bone..

[19] Taylor W, Gladman D, Helliwell P, Marchesoni A, Mease P, Mielants H (2006). Classification criteria for psoriatic arthritis: development of new criteria from a large international study. Arthritis Rheum.

[20] Simon D, Kleyer A, Stemmler F, Simon C, Berlin A, Hueber AJ (2017). Age- and sex-dependent changes of intra-articular cortical and trabecular bone structure and the effects of rheumatoid arthritis. J Bone Miner Res.

[21] Khosla S, Riggs BL, Atkinson EJ, Oberg AL, McDaniel LJ, Holets M (2006). Effects of sex and age on bone microstructure at the ultradistal radius: a population-based noninvasive in vivo assessment. J Bone Miner Res.

[22] Boutroy S, Bouxsein ML, Munoz F, Delmas PD (2005). In vivo assessment of trabecular bone microarchitecture by high-resolution peripheral quantitative computed tomography. J Clin Endocrinol Metab.

[23] van Rietbergen B, Weinans H, Huiskes R, Odgaard A (1995). A new method to determine trabecular bone elastic properties and loading using micromechanical finite-element models. J Biomech.

[24] Pistoia W, van Rietbergen B, Lochmuller EM, Lill CA, Eckstein F, Ruegsegger P (2002). Estimation of distal radius failure load with micro-finite element analysis models based on three-dimensional peripheral quantitative computed tomography images. Bone..

[25] Adamopoulos IE, Chao CC, Geissler R, Laface D, Blumenschein W, Iwakura Y (2010). Interleukin-17A upregulates receptor activator of NF-kappaB on osteoclast precursors. Arthritis Res Ther..

[26] Gravallese EM, Schett G (2018). Effects of the IL-23-IL-17 pathway on bone in spondyloarthritis. Nat Rev Rheumatol.

[27] Lam J, Takeshita S, Barker JE, Kanagawa O, Ross FP, Teitelbaum SL (2000). TNF-alpha induces osteoclastogenesis by direct stimulation of macrophages exposed to permissive levels of RANK ligand. J Clin Invest.

[28] Sato K, Suematsu A, Okamoto K, Yamaguchi A, Morishita Y, Kadono Y (2006). Th17 functions as an osteoclastogenic helper T cell subset that links T cell activation and bone destruction. J Exp Med.

[29] Uehara R, Suzuki Y, Ichikawa Y (2001). Methotrexate (MTX) inhibits osteoblastic differentiation in vitro: possible mechanism of MTX osteopathy. J Rheumatol.

[30] Zhu TY, Griffith JF, Qin L, Hung VW, Fong TN, Au SK (2015). Density, structure, and strength of the distal radius in patients with psoriatic arthritis: the role of inflammation and cardiovascular risk factors. Osteoporos Int.

