# Supplementary material for: Effect of disease-modifying anti-rheumatic drugs on bone structure and strength in psoriatic arthritis patients
Source: Arthritis Res Ther. 2019 Jul 3;21:162. doi: 10.1186/s13075-019-1938-3 (PMC6607518; doi:10.1186/s13075-019-1938-3)
Supplement: Supplementary file 1 — Table S1. Regression models. (DOCX 18 kb) [file 13075_2019_1938_MOESM1_ESM.docx]

Table S1. Regression models

| **Total vBMD** | | | | |
| --- | --- | --- | --- | --- |
|  | **Estimates** | **CI** | **p-Value** | |
| Intercept | 363.03 | 326.19–399.88 | **<0.001** | |
| Age | -1.56 | -2.22 – -0.89 | **<0.001** | |
| Female | 9.65 | -13.22 – 32.52 | 0.406 | |
| bDMARDs | 43.75 | 18.30 – 69.20 | **0.001** | |
| Methotrexate | 11.47 | -18.46 – 41.39 | 0.450 | |
| MDA | -2.74 | -18.90 – 13.43 | 0.739 | |
| **Stiffness** | | | | |
|  | **Estimates** | **CI** | | **p-Value** |
| Intercept | 68.21 | 60.41 – 76.02 | | **<0.001** |
| Age | -0.29 | -0.43 – -0.15 | | **<0.001** |
| Female | -15.95 | -20.83 – -11.07 | | **<0.001** |
| bDMARDs | 6.70 | 1.31 – 12.08 | | **0.015** |
| Methotrexate | -0.19 | -6.66 – 6.29 | | 0.954 |
| MDA | 1.64 | -1.82 – 5.10 | | 0.352 |
| **Failure Load** | | | | |
|  | **Estimates** | **CI** | | **p-Value** |
| Intercept | 3203.37 | 2853.47 – 3553.26 | | **<0.001** |
| Age | -13.27 | -19.61 – -6.93 | | **<0.001** |
| Female | -748.30 | -967.17 – -529.43 | | **<0.001** |
| bDMARDs | 316.38 | 74.99 – 557.76 | | **0.011** |
| Methotrexate | -21.66 | -268.59 – 311.91 | | 0.883 |
| MDA | 89.48 | -65.57 – 244.53 | | 0.256 |

vBMD, volumetric bone mineral density; bDMARDs, biologic disease modifying anti-rheumatic drugs; CI, confidence interval; MDA, minimal disease activity.

Reference for the change is the no treatment group. Models are adjusted for age, gender and treatment gender interaction.
